# An ICCD camera-based time-domain ultrasound-switchable fluorescence imaging system

**DOI:** 10.1038/s41598-019-47156-x

**Published:** 2019-07-22

**Authors:** Shuai Yu, Tingfeng Yao, Baohong Yuan

**Affiliations:** 1 Ultrasound and Optical Imaging Laboratory, Department of Bioengineering, The University of Texas at Arlington, Arlington, TX 76019 USA; 2 Joint Biomedical Engineering Program, The University of Texas at Arlington and The University of Texas Southwestern Medical Center, Dallas, TX 75390 USA

**Keywords:** Biomedical engineering, Ultrasound, Cancer imaging, Fluorescence imaging, Imaging and sensing

## Abstract

Fluorescence imaging in centimeter-deep tissues with high resolution is highly desirable for many biomedical applications. Recently, we have developed a new imaging modality, ultrasound-switchable fluorescence (USF) imaging, for achieving this goal. In our previous work, we successfully achieved USF imaging with several types of USF contrast agents and imaging systems. In this study, we introduced a new USF imaging system: an intensified charge-coupled device (ICCD) camera-based, time-domain USF imaging system. We demonstrated the principle of time-domain USF imaging by using two USF contrast agents. With a series of USF imaging experiments, we demonstrated the tradeoffs among different experimental parameters (i.e., data acquisition time, including CCD camera recording time and intensifier gate delay; focused ultrasound (FU) power; and imaging depth) and the image qualities (i.e., signal-to-noise ratio, spatial resolution, and temporal resolution). In this study, we also discussed several imaging strategies for achieving a high-quality USF image via this time-domain system.

## Introduction

Fluorescence imaging in deep biological tissue is extremely desirable because it reveals the tissue’s structural, functional, and molecular information with high-sensitivity, non-ionizing radiation and good penetration^1–5^. Compared with other imaging modalities such as x-ray, ultrasound, magnetic resonance imaging (MRI), and positron-emission tomography (PET), fluorescence imaging has many unique advantages. One outstanding advantage is its multicolor imaging via multiple fluorophores that thus enables fluorophore-based biomarkers to reveal cellular-level or molecular-level biological interactions^6,7^. Unfortunately, fluorescence imaging suffers from poor spatial resolution (a few millimeters) in opaque biological tissue as a result of high scattering^8^. As a result, fluorescence imaging cannot resolve much interesting microscopic information such as microcirculation, angiogenesis, and cancer metastasis^8–10^ in centimeter-deep tissues.

Over the past few years, several technologies have been proposed and developed to overcome this limitation. One technology is to focus excitation light in deep tissue by focused ultrasound (FU), so called time-reversed ultrasonically encoded optical focusing^11–16^. Another technology is to confine fluorescence emission by focused ultrasound, so called ultrasound modulated or switchable fluorescence^17–42^. The former applies to any fluorophore excitation but requires a complicated imaging system. The latter requires a relatively simple system but demands unique contrast agents. Among them, we have recently developed a technique that belongs to the latter category: ultrasound switchable fluorescence (USF) imaging^17,18,23–29,42^. This technique adopts thermal-sensitive fluorescence contrast agents that can be switched on/off via a FU beam^17,18,23–26^. Fluorophores are quenched due to an initial polar microenvironment in the contrast agents so that they remain in the “off” state when no ultrasound is applied. The fluorophores are “switched-on” when the FU beam is applied. This is because that the tissue temperature is slightly raised beyond a temperature threshold (T_th_) of the contrast agent, which changes the fluorophores’ microenvironment from polar to nonpolar, significantly increasing the fluorophores’ emission efficiency. When the excitation light is on, the emitted fluorescence is detected by the USF imaging system. By scanning the FU transducer, the USF image provides the distribution of the contrast agents in deep tissues with a high spatial resolution (down to hundreds of microns, depending on various experimental parameters).

USF has a similar spatial resolution to other deep-tissue optical imaging technologies developed in the recent years, such as photoacoustic tomography^43,44^, time-reversed ultrasonically encoded optical focusing^11–16^, and ultrasound-modulated fluorescence^21,32,33^. However, USF has many unique features and here we presented a few of them. First, USF signal has high detection specificity to its contrast agent because the detected USF photons only come from the agent. Second, USF has high detection sensitivity because we have developed several USF contrast agents that are highly sensitive to a small temperature change (only a few Celsius degrees) and could release strong fluorescence after being switched on^17,23–26^. Also, USF signal can be externally manipulated by controlling the FU signal and the data acquisition time^27^ in a sensitive USF imaging system, which makes USF achieve a desired signal-to-noise ratio (SNR). Third, since USF only requires a temperature increase of a few Celsius degrees in tissue, it is safe for *in vivo* imaging. The reason is that a USF contrast agent’s temperature threshold (T_th_) could be well controlled slightly above the body temperature (~37 °C) and also its temperature transition bandwidth (T_BW_) could be narrowed down to a few degrees (usually 3–5 °C). As a result, a corresponding temperature increase *in vivo* (e.g., from 37 °C to 40 °C) should provide sufficient energy to acquire a USF signal. Lastly, USF can be inherently combined with high intensity focused ultrasound (HIFU) treatment.

As our previous work has described^23^, we used the following parameters to quantify the performance of a USF contrast: an on-to-off ratio of fluorescence strength (I_on_/I_off_), an on-to-off ratio of fluorescence lifetime (*τ*_on_/*τ*_off_), an adjustable temperature threshold to switch on fluorophores (T_th_), a narrow temperature transition bandwidth (T_BW_), and the fluorophore’s peak excitation (λ_ex_) and peak emission (λ_em_) wavelength, which determines the light penetration depth in a biological tissue. In our previous study^18^, we showed that a contrast agent with a high ratio of *τ*_on_/*τ*_off_ can help to improve SNR by adopting a time-domain system. In this study, we plan to implement this time-domain method via an intensified charge-coupled device (ICCD) camera, which can acquire 2D fluorescence images compared with the previous single point detection via a single channel time-domain system using a photomultiplier tube (PMT). In addition, several experimental parameters (i.e., data acquisition time including CCD camera recording time and intensifier gate delay, FU power, and imaging depth) need to be optimized in the time-domain USF imaging and they can affect image qualities (i.e., SNR, spatial resolution, and temporal resolution).

We demonstrated the working principle of the time-domain USF imaging system, including how the ICCD imaging system measured the fluorescence lifetime, how to acquire a USF signal from fluorescence pulses, and how to scan a USF image in this system. Meanwhile, we introduced two near-infrared (NIR) USF contrast agents for the time-domain USF imaging: ADP(OH)_2_-Bottom^45^ in 5% pluoronic F98 nanocapsule and ZnttbPc in 5% pluoronic F98 nanocapsule. Details of the two fluorophores ADP(OH)_2_-Bottom and ZnttbPc are described in Supplementary Information (Fig. S1). Both nanocapsules have excellent USF properties: excellent I_on_/I_off_, narrow T_BW_, appropriate T_th_, and NIR excitation and emission spectrum. The ADP(OH)_2_-Bottom-based contrast agent has a nearly fixed fluorescence lifetime so that the ratio *τ*_on_/*τ*_off_ is close to 1. Meanwhile, the ZnttbPc-based contrast agent has a high *τ*_on_/*τ*_off_ ratio, which means its fluorescence lifetime significantly increases when the temperature is raised above its T_th_. By comparing these two contrast agents, we have shown how *τ*_on_/*τ*_off_ affects USF signals. Using this ICCD-based time-domain system and the two USF contrast agents, we have investigated how the USF image qualities (i.e., SNR, spatial resolution, and temporal resolution) are affected by the camera’s recording time, FU driving voltage, sample thickness, and intensifier gate delay. In this work, we adopted a silicone phantom model for USF imaging, which is a good choice for a comprehensive analysis of the effects of different parameters on the USF performance.

## Results

### Principles of time-domain fluorescence measurement via a gated ICCD camera

**Figure 1:**
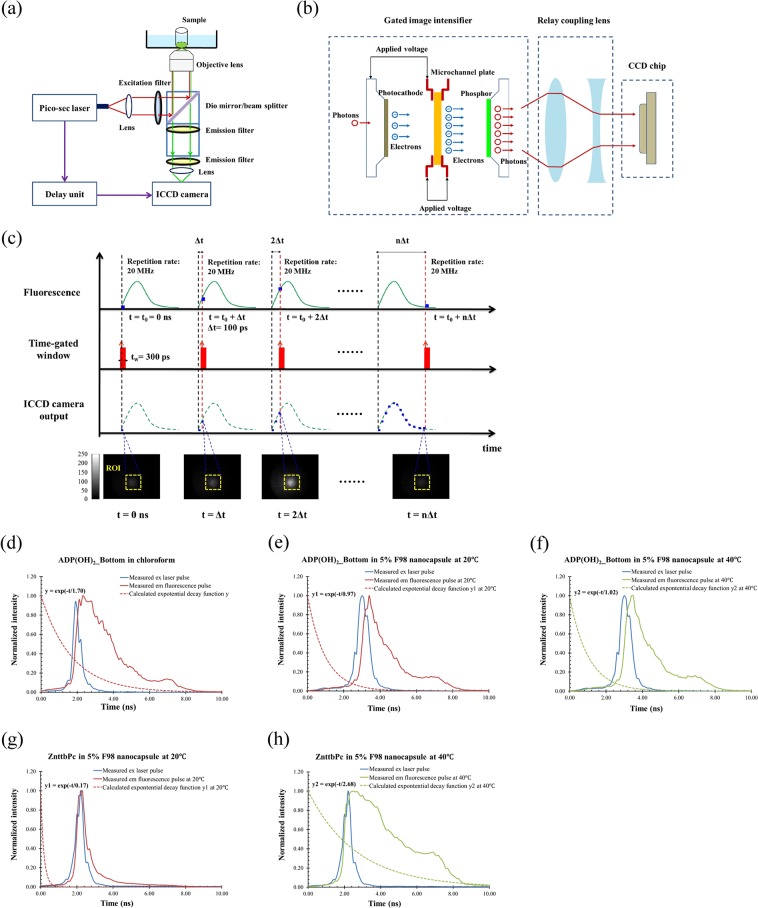
(**a**) Time-domain fluorescence measurement system. (**b**) Schematic diagram of the gated ICCD camera. (**c**) Principle of measuring the fast fluorescence decay signal after excited by a short laser pulse via the gated ICCD camera. (**d**) The measured fluorescence lifetime of the fluorophore of ADP(OH)_2_-Bottom in chloroform. (**e**) The measured fluorescence lifetimes of ADP(OH)_2_-Bottom in 5% pluoronic F98 nanocapsule at 20 °C. (**f**) The measured fluorescence lifetimes of ADP(OH)_2_-Bottom in 5% pluoronic F98 nanocapsule at 40 °C. (**g**) The measured fluorescence lifetimes of ZnttbPc in 5% pluoronic F98 nanocapsule at 20 °C. (**h**) The measured fluorescence lifetimes of ZnttbPc in 5% pluoronic F98 nanocapsule at 40 °C.

When fluorophores are excited by a narrow light pulse, the emission of fluorescence is widely expanded compared with the width of the excitation light pulse (depending upon the fluorescence lifetime of the fluorophores). By measuring this dynamic decay of this fluorescence emission pulse, the lifetime of fluorescence can be quantified. Figure 1(a) schematically shows the measurement system. Briefly, a picosecond (ps) pulsed supercontinuum laser (SC-450, Fianium, Eugene, Oregon; with a broad illumination band) and a time-gated ICCD camera (Picostar HR, LaVision, Goettingen, Germany) were used. The manufacturer-claimed laser pulse width was ~5 ps, and the repetition rate was 20 MHz. The intensifier in ICCD camera was synchronizedly triggered by the laser followed with a delay unit, so that the intensifier was turned on in a selected time-gated window with the same repetition rate (20 MHz). The laser and ICCD camera were coupled into an inverted fluorescence microscope (Ti-U, Nikon). The excitation filter, dichroic mirror/beam splitter and emission filters were selected based on the specific fluorophores (see *Methods* for details). The aqueous fluorescent sample was placed in a quartz cell and submerged in a quartz tank of water on the microscope stage for fluorescence measurement. The temperature of water was controlled and measured by a thermometer for monitoring the environment temperature of the sample.

Figure 1(b) displays a schematic diagram of the gated ICCD camera. Generally, three major parts are included: (1) a gated image intensifier, (2) relay coupling lenses, and (3) a CCD chip. The image intensifier functions as an optical amplifier. Briefly, the incoming photons are first converted into electrons via a photocathode, and then amplified by a short and gated high voltage. After that, the amplified electrons are converted into visible photons via a phosphor. These visible photons are delivered to the CCD chip for imaging via the coupling lens. The temporal resolution of a gated ICCD camera depends on the gate width of the applied high voltage, which, in this study, is down to 300 ps. It is this high temporal resolution that allows an ICCD camera to measure fast fluorescence decay. It is worth mentioning that, compared with the short gate width, the CCD chip usually responds much more slowly and the exposure time is around tens to hundreds of milliseconds. Therefore, the CCD chip is usually used to accumulate those repeated and fast optical signals for achieving an acceptable SNR.

**Figure 2:**
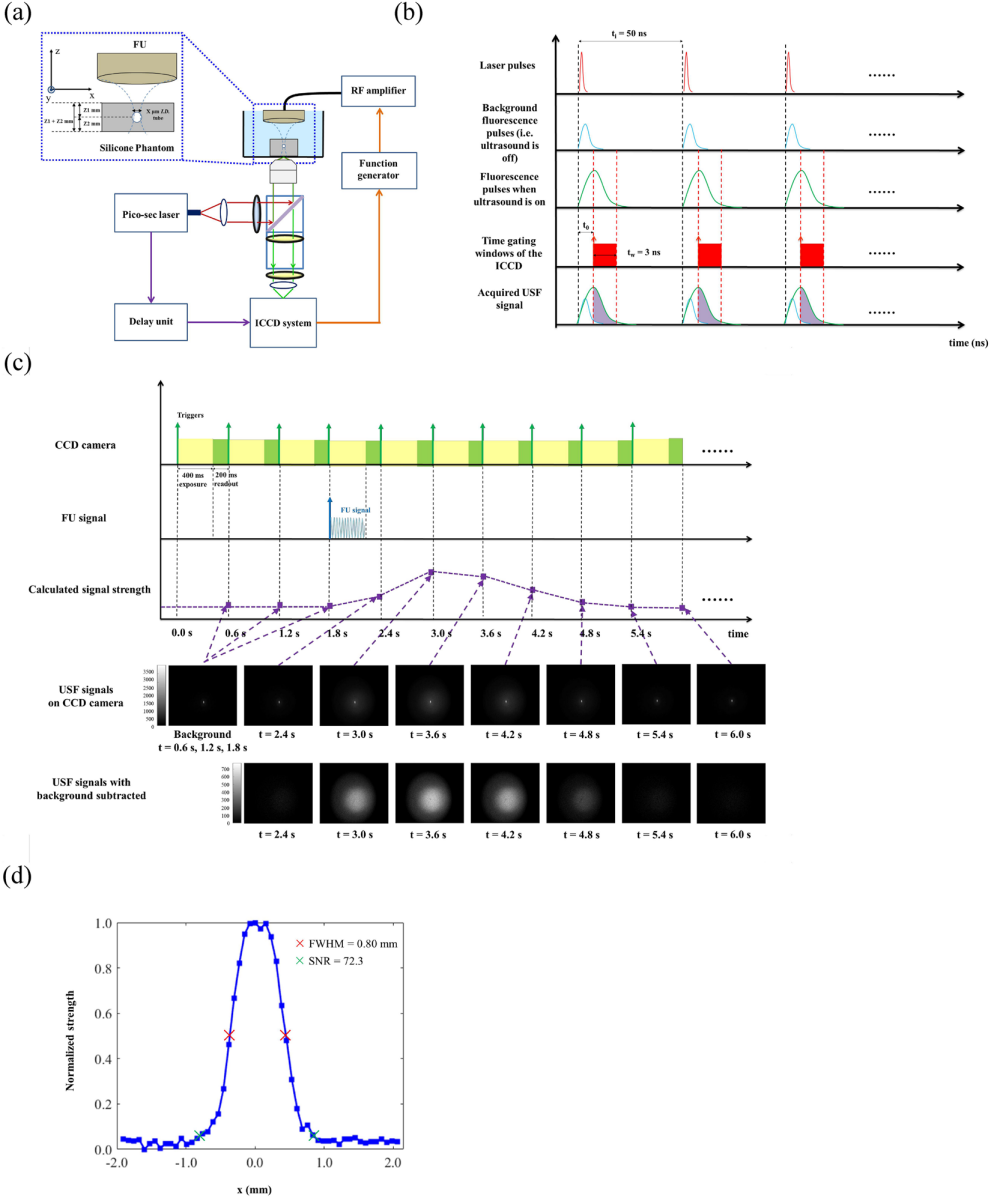
(**a**) Schematic diagram of ICCD camera-based time-domain USF system. (**b**) Time sequences of different events to show how USF signal was acquired at a time scale level of nanoseconds. (**c**) Time sequence of the CCD camera, FU exposure, and acquired USF signals on CCD camera. (**d**) Normalized USF profile of the tube.

To measure the fast fluorescence decay signal after excitation by a short laser pulse via a gated ICCD camera, three steps are followed (see Fig. 1(c)): (1) selecting an appropriate gate width (t_w_ = 300 ps in Fig. 1(c)) for the ICCD camera, fixing the time interval between the laser pulse and the gate voltage of the ICCD’s intensifier, and firing multiple laser pulses (repetition rate: 20 MHz) to accumulate enough photons in the CCD chip (this step provides an image on the CCD chip corresponding to the fluorescence dynamic strength at a single time point (t in Fig. 1(c)); (2) increasing the time interval between the laser pulse and the intensifier gate voltage with a small step (${\rm{\Delta }}t=100$ps in Fig. 1(c)), and repeating step (1); and (3) continuing step (2) until the entire fluorescence decay signal is completely scanned. Thus, a series of images are acquired as a function of the delay time interval (0, ${\rm{\Delta }}t$, 2 ${\rm{\Delta }}t$,…, n ${\rm{\Delta }}t$ in Fig. 1(c)). If an average of the signal strength is calculated for each image within a selected region of interest (ROI), the fluorescence dynamic decay curve can be reconstructed and plotted as a function of time. Figure 1(c) shows an example of a series of recorded images. The blue dot in the third row represents the averaged fluorescence strength in the ROI. It is worth mentioning that because the ICCD camera provides a 2D fluorescence image, this system can measure fluorescence lifetimes at different locations if necessary. A corresponding example is shown in Supplementary Information (Fig. S3).

Figure 1(d–h) show the measured fluorescence lifetimes of the fluorophores used in this study in different formats (fluorophores of ADP(OH)_2_-Bottom in chloroform, ADP(OH)_2_-Bottom encapsulated 5%-F98 pluronic nanocapsule, and ZnttbPc encapsulated 5%-F98 pluronic nanocapsules). All the data are normalized. The blue line in each figure represents the measured excitation laser pulse in each experiment, which represents the system’s impulse response function (IRF). The solid red or green line represents the measured fluorescence pulse. The dashed red or green line represents the calculated exponential decay function, calculated by deconvolving the IRF from the measured fluorescence emission signal (after normalization). The decay factor in the exponential function represents an averaged fluorescence lifetime of each fluorophore. Note that for simplicity we selected an exponential function with a single decay factor. Although this simplification may miss some detailed information when the fluorophore has multiple lifetimes, it is good enough for us to demonstrate the principle of time-domain USF imaging and implement this idea for different fluorophores. Figure 1(d) shows that the fluorescence lifetime of the fluorophore of ADP(OH)_2_-Bottom in chloroform was ~1.70 ns. Figure 1(e–h) show the fluorescence lifetimes of two USF contrast agents: 1) ADP(OH)_2_-Bottom in 5% pluoronic F98 nanocapsule and 2) ZnttbPc in 5% pluoronic F98 nanocapsule at two temperatures: 20 °C and 40 °C. Both USF contrast agents were thermal-sensitive, and their temperature switching thresholds were ~28–31 °C (see Supplementary Information, Fig. S2). Figure 1(e,f) represent the measured fluorescence lifetimes of ADP(OH)_2_-Bottom in 5% pluoronic F98 nanocapsule at 20 °C and 40 °C, respectively. The results indicate that its fluorescence lifetime slightly increased from 0.97 ns to 1.02 ns when temperature rose from 20 °C to 40 °C. If we denote the lifetime as *τ*_off_ when temperature is below the threshold and *τ*_on_ when temperature is above it, the ratio of *τ*_on_ to *τ*_off_ is only 1.05 (i.e., 1.02/0.97). Figure 1(g,h) represent the measured fluorescence lifetime of ZnttbPc in 5% pluoronic F98 nanocapsule at 20 °C and 40 °C, respectively. In contrast, its fluorescence lifetime changed significantly (*τ*_on_/*τ*_off_ = 15.76; i.e., 2.68/0.17). This result was beneficial for USF imaging for achieving high SNR in time domain and was one of the motivations in this study. It is worth mentioning that both USF contrast agents showed a significant increase in fluorescence strength when temperature rose above the threshold. Similarly, if we denote the peak fluorescence strength when temperature is below (T = 20 °C) and above (T = 40 °C) the threshold as I_off_ and I_on,_ respectively, a ratio (I_on_/I_off_) can be calculated. For ADP(OH)_2_-Bottom in 5%-F98 nanocapsules, its I_on_/I_off_ is ~17 folds (I_on_ = 5.74 × 10^3^ counts; the average strength in the selected ROI). For ZnttbPc in 5%-F98 nanocapsules, its I_on_/I_off_ is ~46 folds (I_on_ = 0.55 × 10^3^ counts; the average strength in the selected ROI). To verify these results, we repeated these experiments using a PMT-based fluorescence lifetime measurement system^23^ and achieved similar results (see Supplementary Information, Fig. S2). Therefore, taking advantage of the significant changes in both fluorescence strength and lifetime may help improve USF image quality compared with only using strength change.

### The ICCD camera-based time-domain USF imaging system

By adding an ultrasonic system to the fluorescence lifetime imaging system (i.e., Fig. 1(a)), we can set up an ICCD camera-based time-domain USF system. Figure 2(a) shows the schematic diagram of the system. Compared with Fig. 1(a), a FU transducer (central frequency: 2.5 MHz) and its driving system were added. A function generator (33220 A, Agilent, Santa Clara, CA, USA) was triggered by the ICCD camera via an internal trigger and used to generate the driving signal (i.e., a 2.5 MHz sinusoidal wave). This signal was further amplified by a radiofrequency power amplifier (325LA, E&I, Rochester, NY, USA) and delivered to a FU transducer (H-108, Sonic Concepts Inc, Bothell, WA, USA) to generate an ultrasonic wave. The FU transducer was mounted on a motorized three-dimensional translation stage (Velmex Inc. Bloomfield, NY, USA; not shown in the diagram). Because the whole system was built on the inverted fluorescence microscope, excitation photons were delivered to and emission photons were collected from the bottom of the sample. The ultrasound was delivered from the top and focused inside the sample to generate a temperature rise for switching fluorescence.

Figure 2(a) also shows the configuration of the silicone phantom and the FU transducer (top left). A silicone tube was inserted into the silicone phantom, which was then placed in a quartz tank. The tank was filled with water for coupling the ultrasound wave into the sample and placed on the microscope’s sample stage. TiO_2_ was dissolved in the silicone phantom to make it a scattering medium to mimic biological samples. The reduced scattering coefficient μ_s_’ was 3.5 cm^−1^; the absorption coefficient μ_a_ was 0.03 cm^−1^ ^24,25,46^. The tube in the phantom was filled with USF contrast agents and used as an imaging target. The inner diameter (*I.D*.) and the location of the tube were variable in different experiments. The distance from the top surface of the phantom to the tube center was denoted as Z1 (mm in unit), the distance from the bottom surface of the phantom to the tube center was termed Z2, and the total thickness was (Z1 + Z2). The *I.D*. of the tube was termed X (μm in unit). The details about the sample configuration are described in *Methods* section. For USF imaging, the FU transducer was initially focused on the silicone tube (FU position: x = 0.00 mm, y = 0.00 mm, z = 0.00 mm).

Figure 2(b) displays the time sequences of different events to show how the USF signal was acquired. Note that in this figure the time scale was at a level of nanoseconds, as discussed before. The first row represents the excitation light pulses (the repetition rate was 20 MHz; i.e., the time interval between any two adjacent pulses was t_i_ = 50 ns). The second row indicates the background fluorescence pulses before applying ultrasonic pulses, which had the same repetition rate. The background signal might mainly came from laser leakage, phantom autofluorescence, and background fluorescence from the USF contrast agents that were not 100% off. The third row shows the fluorescence signal after applying ultrasonic pulses (i.e., the sum of USF signal and background signal). The fourth row indicates the gating setup of the ICCD intensifier. Here, the gate was delayed a small amount of time (t_0_) compared with the excitation laser trigger (i.e., the time interval between the black and the first red dash lines) to avoid acquiring the background fluorescence but to acquire the USF signal roughly starting from the peak of the pulse. The gating width was 3 ns with the 20 MHz repetition rate. Thus, only the photons within the gate could be detected. The fifth row indicates the acquired USF signal in the gated window. Theoretically, the USF signal in each gate can be extracted by subtracting the background signal from the total signal (the shadowed area). Practically, this subtraction was not conducted in each individual gate. Instead, depending on the CCD exposure time (at a level of hundreds of milliseconds in this study), the subtraction was conducted between two CCD images that have accumulated many pulses (i.e., the CCD image without ultrasound exposure was subtracted from the other one with ultrasound exposure; see Fig. 2(c)). Depending on how fast the curves would decay in the background and USF signals, varying the delay time t_0_ may optimize the SNR of USF imaging, which was investigated in the following sections.

Figure 2(c) shows the time sequence of the CCD camera, FU exposure, and acquired USF signals on the CCD camera. The CCD camera continuously took frames with a fixed exposure time of 400 ms (see the light yellow region) and a readout time of 200 ms (see the light green region). Before the FU was triggered, the camera acquired several frames (here, number of frames = 3) as the background baseline. Then, the FU transducer was triggered on via the internal trigger from the CCD camera (t = 1.8 s). The FU exposure duration was also 400 ms. The driving peak-to-peak voltage from the function generator was 130 mV and was amplified with a 50 dB gain via the power amplifier. As an example, a series of CCD frames acquired at different times (t = 0.6 s, 1.2 s, … 6.0 s) are shown in the figure. The silicone phantom adopted in this experiment had a thickness of 12 mm (Z1 = 6 mm, Z2 = 6 mm) and a tube *I.D*. X = 310 μm. ADP(OH)_2_-Bottom encapsulated 5%-F98 pluronic nanocapsule solution was filled in the tube as the USF contrast agent. This USF signal was acquired when the FU was focused on the tube (FU position x = 0.00 mm, y = 0.00 mm, z = 0.00 mm). To extract the USF signal, one background frame acquired at t = 1.8 s (which was right before the FU signal) was subtracted from each frame acquired after the FU exposure (i.e., the frames acquired at t = 2.4 s, 3.0 s, … 6.0 s). The figure also shows the resulting frames (last row). To represent the USF signal strength as a single value, we adopted the simple approach of summing up the counts of all the pixels in each frame. The purple squares in the third row represent the calculated USF strength as a function of time using this method.

Because of scattering, the USF photons displayed on the CCD camera frames were spatially scattered as a spot. The full width at half maximum (FWHM) of the spot here was ~2.0 mm, which was much larger than the tube *I.D*. (X = 310 μm) and the lateral focal size of the FU transducer at half-amplitude (~0.8 mm). In addition, the purple dash line in Fig. 2(c) clearly shows that the FU exposure can induce USF photons. After the FU exposure, USF signal strength reached the maximum. After that, the signal strength gradually decreased. This procedure can be understood based on the following mechanism. The FU exposure led to the temperature rising in the ultrasound focal volume. The USF agents in this volume where the temperature was above the switching threshold (T_th_ = 31 °C) were switched on to emit fluorescence. This volume expanded after the FU exposure, then gradually decreased and eventually vanished.

By scanning the FU transducer focus and acquiring USF signals at each location, a USF image was formed. Figure 2(d) shows the normalized USF profile of the tube. The FU transducer scanned across the tube along the x-axis with a total scanning range of 3.96 mm and a step size of 76.2 μm (i.e., 53 scanning points along the x-axis). Here, we counted the USF signal at t = 2.4 s with background subtracted as the signal strength. The FWHM of the USF profile is 0.80 mm, when the tube *I.D*. is 0.31 mm. The SNR is 72.3. To calculate SNR, the peak USF strength was divided by the standard deviation of the background strength. The background region was selected as those data points that were far away from the peak signal (i.e., the data points on the left of the left green cross and those on the right of the right green cross in Fig. 2(d)). The same rule was adopted for other examples in this study.

### The effect of CCD camera recording time on USF imaging

**Figure 3:**
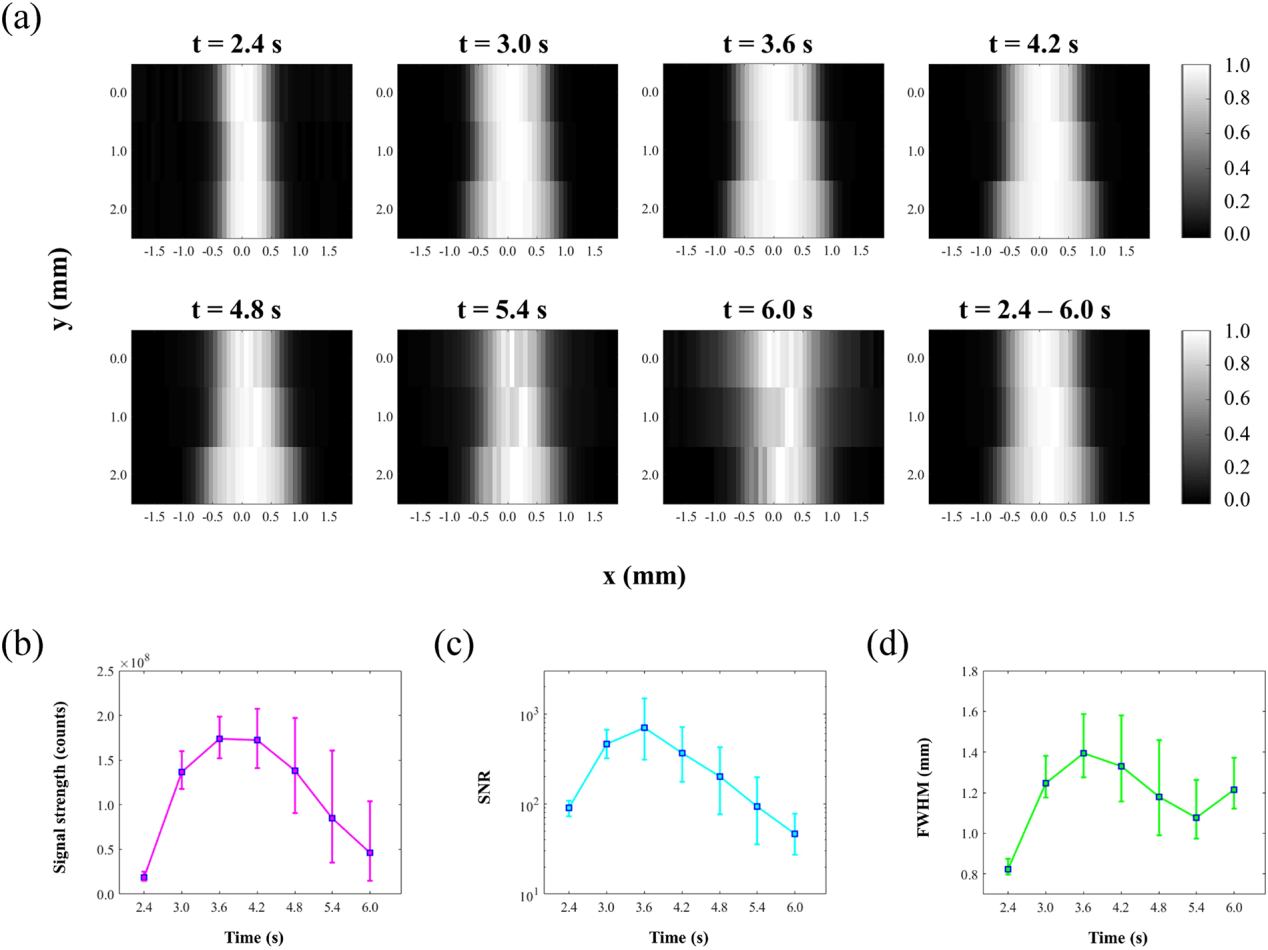
(**a**) USF images of the silicone tube when counting USF signal from different CCD camera recording time t = 2.4 s, 3.0 s, … 6.0 s. (**b**) Relationship between USF signal strength acquired on the center of the tube and CCD camera recording time. (**c**) Relationship between the signal-to-noise ratio (SNR) of USF images and CCD camera recording time t. (**d**) Relationship between the full width at half maximum (FWHM) of imaged tube and CCD camera recording time t.

When the FU transducer scanned along both x-axis and y-axis, a 2D USF image was acquired. Following the previous section, we adopted the same silicone phantom (Z1 = 6 mm, Z2 = 6 mm, X = 310 μm) for 2D USF imaging. The FU transducer scanned across the tube along the x-axis at three y-axis positions (y = 0.00 mm, 1.01 mm, 2.03 mm). Likewise, we counted the USF signal at t = 2.4 s with background subtracted as the signal strength. The image in the first row and the first column in Fig. 3(a) shows the corresponding normalized 2D USF image. The average FWHM of the USF image of the tube is 0.82 mm, and the average SNR is 89.9. Other images in Fig. 3(a) represent the USF images of the same tubes but we counted the USF signal from other frames (i.e., t = 3.0 s, 3.6 s, … 6.0 s) with background subtracted. Here, the time of t indicates the CCD camera recording time. On each USF image in Fig. 3(a), we calculated the average of the three USF signal strengths acquired at the center of the tube (i.e., at x = 0.00 mm, while y = 0.00 mm, 1.01, and 2.03 mm) and plotted them as a function of time t in Fig. 3(b). The blue squares represent the average strength, and the error bar represents the upper and lower values. The plot shows that the signal strength increases first and then decreases, which agrees with the result shown in Fig. 2(c). Similarly, Fig. 3(c) shows the relationship between the averaged SNR and t. SNR reaches 89.9 when t = 2.4 s and increases to 700.0 when t = 3.6 s. After that, SNR gradually decreases to 46.6 when t = 6.0 s. Figure 3(d) shows the relationship between the FWHMs of the USF images in Fig. 3(a) and t. At t = 2.4 s, FWHM is as small as 0.82 mm, which represents the highest spatial resolution. When t = 3.6 s and 6.0 s, FWHM degrades to 1.40 mm and 1.21 mm, respectively.

Figure 3(b,c) indicate that a higher signal strength provided a higher SNR of image, because background noise should all be similar in this experiment. Figure 3(b–d) indicate that USF signal strength, SNR, and FWHM increase first and then decrease. This means that adopting an early frame (t = 2.4 s) or a late frame (t = 5.4 s or 6.0 s) for calculating the USF strength enables achievement of a relatively higher spatial resolution at the expense of signal strength and SNR. On the other hand, adopting a middle frame (t = 3.6 s) enables achievement of a relatively higher signal strength and SNR at the expense of spatial resolution. This tradeoff consists with the fact that a larger voxel can generate a stronger signal but provide lower spatial resolution. On the other hand, increasing the CCD exposure time over the entire signal duration can help to collect more USF photons to increase the signal strength and SNR, but may degrade the spatial resolution because of possible thermal diffusion. This has been demonstrated in the last image in Fig. 3(a). This image is the mathematical sum of all images from t = 2.4 s to t = 6.0 s and gives a relatively high SNR (245.3) but a relatively large FWHM (1.26 mm). These results indicate that SNR and spatial resolution depend on the CCD camera recording time when the USF signal is acquired and that these two parameters may need to be balanced.

### The effect of FU driving voltage on USF images

In this section, we studied the relationship between FU driving voltage and imaging qualities (i.e., signal strength, SNR, and resolution). Theoretically, the square of the driving voltage of the FU transducer is proportional to the ultrasound exposure power. In the experiments, it was difficult to quantify the actual power in the focal area. Therefore, we selected the FU driving voltage from the function generator as the experimental variable. The same silicone phantom was adopted (Z1 = 6 mm, Z2 = 6 mm, X = 310 μm) for USF imaging. ADP(OH)_2_-Bottom encapsulated 5%-F98 pluronic nanocapsule solution was filled in the tube as the USF contrast agent. Similarly, the FU transducer scanned across the tube (i.e., along the x-axis) with a total scanning range of 3.05 mm and a step size of 76.2 μm (i.e., 41 scanning points along the x-axis) to achieve a line scan at one FU driving voltage. The same line scan was then conducted with a different FU driving voltage. In this experiment, the driving peak-to-peak voltages (Vpps) were 70 mV, 100 mV, 130 mV, and 160 mV, respectively. All other experimental parameters and data processing remained the same as in the previous experiment.

**Figure 4:**
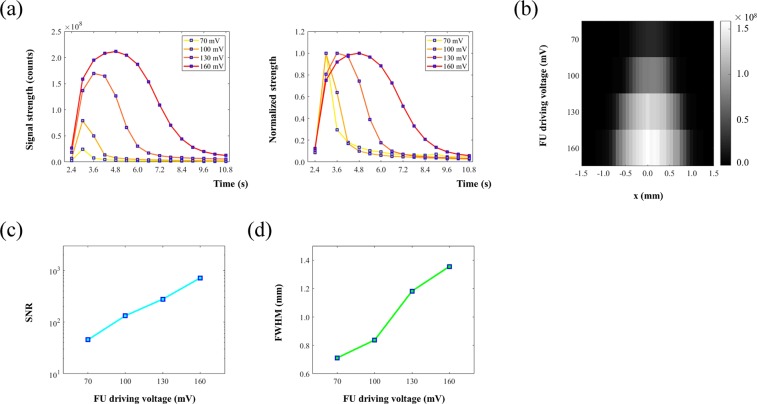
(**a**) The USF signals acquired at x = 0.00 mm (i.e., FU was focused on center of the tube) with different Vpp (i.e., Vpp = 70 mV, 100 mV, 130 mV and 160 mV). In the left plot, it shows the USF signal strength increased with Vpp. In the right plot, it shows the normalized signal strengths over time: USF signal duration also increased with the increase of Vpp. (**b**) 2D USF image representing the relationship between image profile and FU power. (**c**) Relationship between SNR of imaged tube and FU power. (**d**) Relationship between FWHM of imaged tube and FU power.

Figure 4(a) shows the USF signals acquired at x = 0.00 mm (i.e., FU was focused on the center of the tube) with different Vpp (i.e., Vpp = 70 mV, 100 mV, 130 mV, and 160 mV). The left plot shows that the USF signal strength increased with Vpp. Peak signal strengths were 0.25, 0.79, 1.70, and 2.12 (×10^8^) counts, respectively. The right plot shows the normalized signal strengths over time, respectively. It shows the USF signal duration also increased with the increase of Vpp. The rise time was 0.42 s, 0.41 s, 0.97 s, and 2.16 s, and the fall time was 0.54 s, 0.94 s, 1.86 s, and 2.87 s, respectively. The definition of rise or fall time here is the time duration from 37% to 100% signal strength increase or from 100% to 37% signal strength decrease. This agrees with the fact that the ultrasound-heated volume in which the temperature was above the switching threshold of the agent increases with the Vpp (i.e., FU exposure power).

A 2D image was plotted to further show the effect of the Vpp on USF signal in Fig. 4(b). USF signals acquired at the frame t = 3.0 s (with background subtracted) were used to represent the strength. The horizontal axis represents the FU scanning range across the tube (i.e., along the x-axis at y = 0.00 mm). The vertical axis represents the corresponding Vpp. The maximum USF strength at each Vpp was 0.26, 0.82, 1.37, and 1.59 (×10^8^) counts, respectively. It is worth mentioning that the maximum USF strength here was not necessarily the same as the peak strength in Fig. 4(a) because (1) the maximum USF strength here was not necessary at x = 0.00 mm, and (2) the USF strength acquired here at the frame t = 3.0 s was not necessary at the same frame when USF signal reached peak: Fig. 2(a) shows USF signal reached peak at frame t = 3.0 s, 3.0 s, 3.6 s, and 4.8 s when Vpp = 70 mV, 100 mV, 130 mV, and 160 mV, respectively. At the same time, as the Vpp rose, the USF image of the tube broadened. Figure 4(c,d) represent the corresponding changes of SNR and FWHM at the four driving voltages. For the four Vpps, the SNR is 45.5, 133.0, 277.1, and 712.7, and the FWHM is 0.71 mm, 0.84 mm, 1.18 mm, and 1.36 mm, respectively. This indicates that increasing Vpp can improve SNR but degrade spatial resolution. Accordingly, SNR and spatial resolution may need to be balanced when selecting appropriate Vpp.

### The effect of the sample thickness on USF imaging

**Figure 5:**
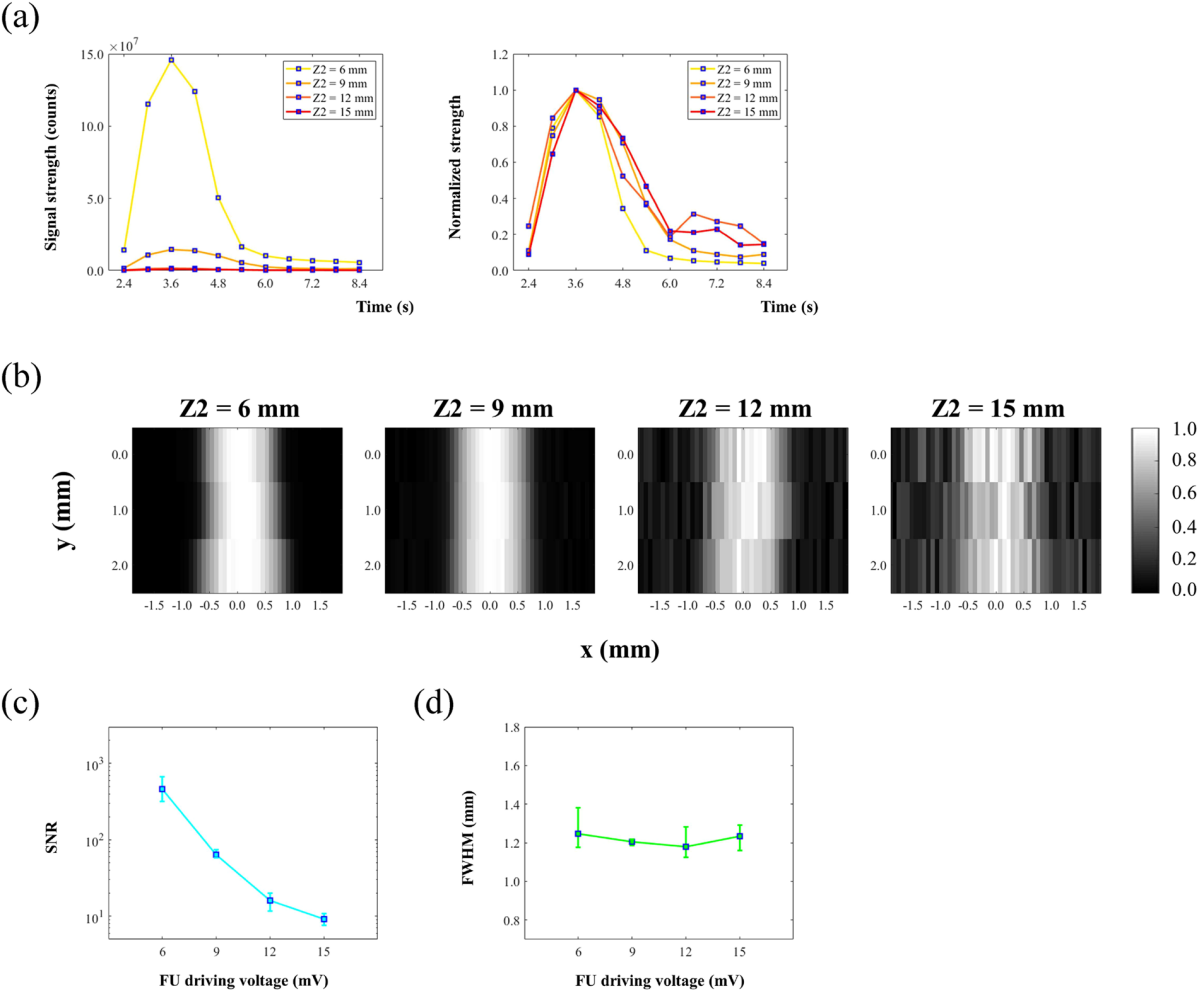
(**a**) USF signals acquired at x = 0.00 mm, y = 0.00 mm, in the four phantoms with different Z2 = 6, 9, 12 and 15 mm. In the left plot, it shows the USF signal strength significantly decreased with the increase of Z2. In the right plot it shows the normalized signal strengths over time: the USF signal duration changed slightly when the Z2 increased. (**b**) Normalized 2D USF images acquired at different imaging depth Z2 = 6, 9, 12 and 15 mm. (**c**) Relationship between SNR of imaged tube and imaging depth Z2. (d) Relationship between FWHM of imaged tube and imaging depth Z2.

This section discusses the relationship between sample thickness and imaging qualities (i.e., signal strength, SNR, and resolution). In this experiment, we made four phantoms with different thicknesses. Specifically, Z2 varied from 6, 9, 12 to15 mm, respectively, but Z1 remained at 6 mm, and the *I.D*. of the tube remained at 310 μm for all the Z2. The Vpp was 130 mV. In the 2D USF imaging of each phantom, the FU transducer scanned across the tube along the x-axis with a total scanning range of 3.96 mm and a step size of 76.2 μm (i.e., 53 scanning points along the x-axis) at three y-axis positions (y = 0.00 mm, 1.01 mm, 2.03 mm). Other experimental parameters remained the same as before. Figure 5(a) represents the USF signals acquired at x = 0.00 mm, y = 0.00 mm, in the four phantoms with Z2 = 6, 9, 12, and 15 mm, respectively. The left plot shows that the USF signal strength significantly decreased as Z2 increased. Their peak strengths were 14.58, 1.45, 0.15, and 0.09 (×10^7^) counts, respectively. Meanwhile, the right plot displays the respective normalized signal strengths over time and shows that the USF signal duration changed slightly when Z2 increased. The rise time was 0.96 s, 0.96 s, 1.08 s, and 0.90 s, and the fall time was 1.17 s, 1.79 s, 1.81 s, and 2.03 s, respectively.

Figure 5(b) shows the normalized 2D USF images acquired at different Z2. Here, we used USF signals acquired at frame t = 3.0 s (with background subtracted) to represent the signal strength. The results also indicate that the SNR decreased very quickly when Z2 increased. However, the imaged tube did not obviously broaden or narrow (i.e., the FWHM remained stable). Figure 5(c,d) represent the relationships of SNR and FWHM with Z2, respectively. The blue square represents the average strength, and the error bar represents the upper and the lower values. The SNR = 459.0, 63.9, 16.0, and 9.2 (average value), and the FWHM = 1.25 mm, 1.21 mm, 1.18 mm, and 1.23 mm when, correspondingly, Z2 = 6, 9, 12 and 15 mm.

The results show that the SNR decreased quickly (Fig. 5(c)) as the sample thickness increased. This is understandable because the thicker the sample was, the more excitation and emission photons were lost due to the scattering and absorption. However, the signal duration (the right plot in Fig. 5(a)) and the FWHM (Fig. 5(d)) appeared independent of the thickness. This is because they were mainly determined by the size of the ultrasound-heated volume where the temperature was above the switching threshold of the agent, which was independent of Z2 in this study. Note that the FU Vpp in this experiment remained at 130 mV, and the ultrasound penetration depth remained at Z1 = 6 mm.

### The effect of gating delay on USF images

In the ICCD camera-based, time-domain USF imaging system, the gate delay of the ICCD camera relative to the laser pulse can be well controlled. This capability provides a way to further improve SNR based on the following assumptions. First, the laser leakage and the sample autofluorescence usually have shorter lifetimes compared with that of the USF signal. Thus, they may be removed by appropriately delaying the intensifier gate. If the gate is delayed to a time point when the laser leakage and autofluorescence are low (because of their short lifetimes) but the USF signal is still strong (because of its long lifetime), it is possible to have a higher signal-to-background ratio (SBR) and therefore a higher SNR. Second, when a USF contrast agent has a high ratio of *τ*_on_/*τ*_off_, appropriately delaying the gate may also potentially reduce the background noise generated by non-100% off USF contrast agents. When the USF contrast agent is not 100% off, it may emit some fluorescence even without ultrasound. If the fluorescence lifetime of this background noise (denoted as *τ*_off_) is much shorter than that of the ultrasound-switched on contrast agents (denoted as *τ*_on_), the gating method may be very helpful to remove this type of noise as well. The new USF contrast agent of ZnttbPc encapsulated 5%-F98 pluronic nanocapsules has shown a high value of *τ*_on_/*τ*_off_.

**Figure 6:**
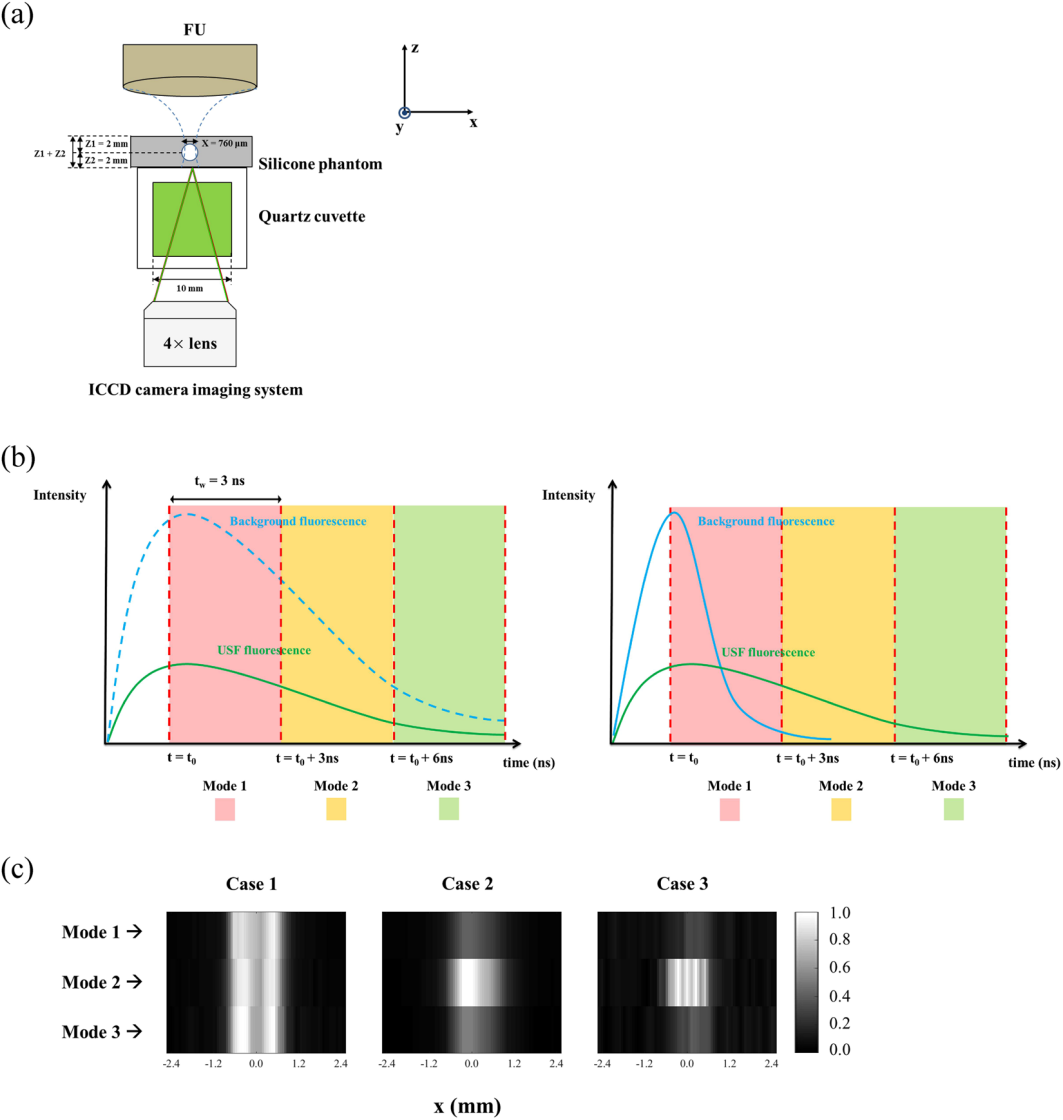
(**a**) Silicone phantom and USF signal measurement setup. (**b**) Scheme of the three selected time-gated window (i.e., mode 1, mode 2, and mode 3) for the three cases: the left panel represents Case 1 in which both the background and the USF signal had similar lifetimes and decayed at a similar speed; the right panel represents Case 2 and 3 in which the background had a short lifetime and decayed fast while the USF signal had a long lifetime and decayed slowly. (**c**) USF images acquired at the three modes for the three cases.

To demonstrate the above ideas via the time-domain system, a new phantom was set up, as Fig. 6(a) shows. We adopted a silicone phantom with a thickness of 4 mm for USF imaging. Other parameters were similar to the ones used before. A silicone tube with an *I.D*. X = 760 μm was embedded in the middle of the phantom and filled with one of the two types of USF contrast agents (see the details in Table 1). For USF imaging, the FU transducer was initially focused on the silicone tube (FU position: x = 0.00 mm, y = 0.00 mm, z = 0.00 mm). The contrast agents in the tube were considered as the imaging target. A quartz cuvette was placed under the silicone phantom and filled with one of the two types of USF contrast agents (see the details in Table 1). Thus, the contrast agents in the cuvette generated a strong fluorescence emission that was considered background noise. Three combinations of different USF contrast agents were filled in the silicone tube and the cuvette.

**Table 1:** NC: Nanocapsules.

	Tube (Target: *τ*_on_)	Cuvette (Background: *τ*_off_)	*τ*_on_/*τ*_off_ (T/B)
Case 1	ADP(OH)_2_-Bottom NC (*τ*_on_ = 1.02 ns)	ADP(OH)_2_-Bottom NC (*τ*_off_ = 0.97 ns)	1.05 (=1.02/0.97)
Case 2	ADP(OH)_2_-Bottom NC (*τ*_on_ = 1.02 ns)	ZnttbPc NC (*τ*_off_ = 0.17 ns)	6.00 (=1.02/0.17)
Case 3	ZnttbPc NC (*τ*_on_ = 2.68 ns)	ZnttbPc NC (*τ*_off_ = 0.17 ns)	15.76 (=2.68/0.17)

In Case 1, both the tube and the cuvette were filled with ADP(OH)_2_-Bottom encapsulated 5%-F98 pluronic nanocapsules. In Case 2, the tube was filled with ADP(OH)_2_-Bottom encapsulated 5%-F98 pluronic nanocapsules and the cuvette with ZnttbPc encapsulated 5%-F98 pluronic nanocapsules. In Case 3, both the tube and the cuvette were filled with ZnttbPc encapsulated 5%-F98 pluronic nanocapsules. For ADP(OH)_2_-Bottom based contrast agent: *τ*_on_ = 1.02 ns and *τ*_off_ = 0.97 ns. For ZnttbPc based contrast agent: *τ*_on_ = 2.68 ns and *τ*_off_ = 0.17 ns.

Based on Fig. 1(e,f), the ADP(OH)_2_-Bottom-based agent had a fluorescence lifetime of *τ*_off_ = 0.97 and *τ*_on_ = 1.02 ns, respectively, which gave a ratio of *τ*_on_/*τ*_off_ = 1.05. On the other hand, the ZnttbPc-based agent had a lifetime of *τ*_off_ = 0.17 and *τ*_on_ = 2.68 ns, respectively, which gave a ratio of *τ*_on_/*τ*_off_ = 15.76. In Case 1, the ADP(OH)_2_-Bottom-based agent was in both the target and the background, and *τ*_on_/*τ*_off_ ≈ 1, which simulated a situation in which the target had a lifetime similar to that of the background. This scenario may occur when the adopted USF agent does not change its fluorescence lifetime much before and after being switched on (i.e., only the emission strength increases). In Case 2, the ZnttbPc-based agent was in the background and was not switched on. Therefore, *τ*_off_ = 0.17 ns was considered the background fluorescence lifetime. In contrast, the ADP(OH)_2_-Bottom-based agent was in the target and was switched on by the ultrasound. Accordingly, *τ*_on_ = 1.02 ns was considered the target fluorescence lifetime. Thus, Case 2 mimicked a situation of *τ*_on_/*τ*_off_ = 6.00 (i.e., 1.02/0.17), meaning that the target had a relatively longer lifetime than the background. This scenario may occur when the laser leakage or sample autofluorescence dominates the background noise. Case 3 had a ratio of *τ*_on_/*τ*_off_ = 15.76, which mimicked a situation in which the target had a much longer lifetime than that of the background. This scenario may occur when the background noise is mainly generated from the non-100% off USF agents. Accordingly, we expect that the method of delaying the gate may affect the SNR in Case 2 and 3 but may not in Case 1.

Figure 6(b) schematically shows three modes of delaying the imaging gate for the three different cases. Note that the time scale was at a level of nanoseconds here (similar to Fig. 2(b)). The left panel represents Case 1, in which both the background and the USF signal had similar lifetimes and decayed at a similar speed. The right panel represents Case 2 and 3, in which the background had a short lifetime and decayed fast while the USF signal had a long lifetime and decayed slowly. Mode 1 indicates that the gate was slightly delayed compared with the laser pulse so that the fluorescence emission peak was located within the gate. Mode 2 means that the gate was delayed 3 ns (in this study) more than Mode 1. Mode 3 was delayed 3 ns more than Mode 2 (i.e., 6 ns more compared with Mode 1). The gate width was 3 ns in all three modes.

Figure 6(c) shows the USF images acquired at the three modes for the three cases. In all USF images, FU was scanned across the tube (i.e., along the x-axis) with a total scanning range of 5.08 mm and a step size of 127.0 μm (i.e., 41 scanning points). The Vpp was 130 mV. To make the USF images directly comparable, the ICCD camera intensifier gain in each experiment was tuned to keep the background signal at the same level. To increase the sensitivity, the CCD camera exposure time was increased to 3 seconds so that the USF signal was acquired in one frame after FU exposure, an idea discussed in the previous section (represented in the last image in Fig. 3(a)). The data were linearly interpolated to achieve smooth images. All other experimental parameters remained the same. Table 2 summarizes the SNR and FWHM of the USF images. From both this table and the images in Fig. 6(c), we find that in Case 2 and 3 the USF images achieved the highest SNRs when Mode 2 was adopted. This result validates the idea that the SNR can be optimized by adjusting the delay time of the ICCD’s gate when the target’s fluorescence lifetime is longer than that of the background noise. On the other hand, in Case 1, all three modes provided USF images with similar SNRs. This result validates the idea that the method of adjusting the delay time of the ICCD’s gate does not increase the SNR when the target’s lifetime is similar to that of the background noise. Meanwhile, the FWHMs of the imaged tube in the three modes remained similar in each case (see Table 2), which indicates that spatial resolution was independent of gating delay.

**Table 2:** In Case 1, Mode 1–3 provided USF images with similar SNRs; in both Case 2 and Case 3, USF images achieved the highest SNRs when the Mode 2 was adopted.

	Case 1		Case 2		Case 3	
	SNR	FWHM (mm)	SNR	FWHM (mm)	SNR	FWHM (mm)
**Mode 1**	46.4	1.48	37.1	1.31	7.8	0.76
**Mode 2**	47.3	1.46	**53.7**	1.25	**34.1**	1.11
**Mode 3**	47.5	1.45	36.3	1.22	8.0	0.98

In all cases, FWHMs remained similar in the three modes.

Note that the switched-on fluorescence lifetimes (*τ*_on_) discussed here and shown in Fig. 1(f,h) were measured by immersing the USF agents in a hot water bath with a temperature above the switching threshold of the agent (i.e., T = 40 °C > T_th_). We also developed a method to directly measure the *τ*_on_ when using ultrasound to switch on the agent (instead of using the hot water bath). Fig. S4 (see Supplementary Information) shows the measurement methods and results. The results indicate that the measured switched-on fluorescence lifetimes (*τ*_on_) via the two methods were similar.

## Discussion

### Experimental parameters vs. USF image quality

Based on the above results, we summarize how the experimental parameters affected the USF image qualities (signal strength, SNR, spatial resolution, and temporal resolution) in Table 3. Clearly, the increase (or optimization) of all the listed experimental parameters (except the sample thickness) can improve (or optimize) the signal strength and SNR, which is helpful to achieve high-quality USF images. However, care must be taken when manipulating these parameters because most of them may lead to degradation of spatial resolution (except for the gating delay and the sample thickness). The same conclusion obviously holds for the temporal resolution, although we have not discussed it much here. These experimental parameters can be classified into three groups: optical, acoustic, and sample-related parameters, all of which are either directly adjustable or related to specific experiments and therefore must be balanced or optimized to achieve a desired USF image.

**Table 3:** Exp. Para.: experimental parameters; Imag. Para.: image parameters; Sig. Str.: signal strength; Spa. Resl.: spatial resolution; Temp.Resl.: temporal resolution. ↑: increase; ↓: decrease; ↑↓: increase and then decrease; ↓↑: decrease and then increase; ×: not correlated.

Img. Para.	Sig. Str. & SNR	Spa. Resl.	Temp. Resl.
Exp. Para.			
(optical) CCD camera recording time ↑	↑↓	↓↑	↓
(acoustic) FU power ↑	↑	↓	↓
(sample) Thickness ↑	↓	×	×
(optical) Gating delay ↑	↑↓ or ×	×	×
(optical) Total CCD exposure time ↑	↑	↓	↓

### Selecting appropriate USF contrast agents

Besides balancing and optimizing different experimental parameters, selecting appropriate USF contrast agents is also important in time-domain USF imaging. An excellent time-domain USF contrast agent may include high on-to-off ratios of both fluorescence strength (I_on_/I_off_) and lifetime (*τ*_on_/*τ*_off_) as well as USF signal strength itself (I_on_). Similar to other USF imaging modes (such as continuous wave and frequency domain modes), an adjustable switching temperature threshold (T_th_), a narrow temperature transition bandwidth (T_BW_), and near-infrared excitation and emission wavelength (λ_ex_ and λ_em_) are also desirable. These factors may be considered together rather than singly. For example, for time-domain USF imaging, I_on_/I_off_, *τ*_on_/*τ*_off_ and I_on_ should be considered together. We have demonstrated that the ZnttbPc-based agent has a high ratio for both I_on_/I_off_ (=~46 folds) and *τ*_on_/*τ*_off_ (=15.76). Thus, the ZnttbPc-based agent is suitable for time-domain USF imaging because the background noise can be efficiently reduced via the gating delay method. However, this does not mean that the ZnttbPc-based agent would be a better choice than the ADP(OH)_2_-Bottom-based agent in all cases. Although the ADP(OH)_2_-Bottom-based agent has a relatively lower I_on_/I_off_ (=~17 folds) and a slightly changed lifetime (*τ*_on_/*τ*_off_ = 1.05), it has a higher I_on_ than that of the ZnttbPc-based agent (I_on_: 5.74 × 10^3^ counts >0.55 × 10^3^ counts, based on the results in the previous section). Thus, the ADP(OH)_2_-Bottom-based agent has a higher detection sensitivity. Although it may not benefit much for time-domain USF imaging, the ADP(OH)_2_-Bottom-based agent is a good choice in those cases where the USF signal strength might be highly attenuated and hard to detect, such as in deep biological tissue.

### Additional discussions for current USF imaging

The current USF system was built on a fluorescence microscope. The adopted objective lens was a 4× Nikon lens, which limited the field of view (FOV) to ~3.2 mm in diameter. This FOV was relatively small. If the USF signal comes from a sample with a thickness of a few millimeters or the scattering is not significant, this FOV may be enough to collect most USF photons emitted from the sample surface (see the USF scattering spot in Fig. 2(c)). However, when the USF photons are coming from a much thicker sample and/or the scattering is significant, a larger FOV should be adopted for maximal coverage of the USF scattering area, which would therefore increase collection efficiency. This can be achieved by adopting a new lens system and will be investigated in future.

Another consideration regarding time-domain USF imaging is the possible temporal expansion of the excitation and emission pulses caused by sample scattering effect. For example, although the ZnttbPc-based agent’s fluorescence lifetime significantly increases from hundreds of picoseconds to a few nanoseconds after the fluorophores are switched on, the emission pulse will eventually be convolved with the optical impulse response function of the scattering medium after passing throughout. Thus, the emission pulse might broaden to a level when *τ*_on_/*τ*_off_ becomes low, which matters especially when implementing USF imaging in centimeter-deep biological tissue. How this scattering effect affects the gating method should be investigated in future. If this effect is significant, this potential issue may be addressed in two ways: (1) develop new USF contrast agents that not only have a high *τ*_on_/*τ*_off_ but also have a large *τ*_on_; (2) use a model to quantify and/or deconvolve this scattering effect.

## Conclusion

In this work, we introduced the ICCD camera-based, time-domain USF imaging system. By implementing USF experiments on this system, we demonstrated its features and advantages for USF imaging. We also described the tradeoffs inherent in USF imaging and suggested appropriate USF imaging strategies, including balancing the tradeoffs by changing experimental parameters (CCD camera recording time, FU power, imaging depth, and gate delay of ICCD camera) and selecting an appropriate USF contrast agent.

## Methods

### Chemical materials

Tetrabutylammonium iodide (TBAI), zinc 2,9,16,23-tetra-tert-butyl-29H,31H-phthalocyanine (ZnttbPc), and chloroform was purchased from Sigma-Aldrich Corporate (St. Louis, MO, USA). Pluronic F98 pastille was purchased from BASF Corporation (Vandalia, IL, USA). BF2-chelated [5-(4-hydroxyphenyl)-3-phenyl-1H-pyrrol-2-yl]-[5-(4-hydroxyphenyl)-3-phenylpyrrol-2-ylidene] amine (ADP(OH)_2_-Bottom)^45^ was synthesized at the Department of Chemistry, University of North Texas (Denton). All chemicals were used as received without further purification.

### Optical filter set-up in ICCD camera imaging system

The optical filter sets (Semrock, Rochester, New York) for fluorescence pulse measurement of the samples (i.e., ADP(OH)_2_-Bottom dye in chloroform, ADP(OH)_2_-Bottom in 5% F98 nanocapsule, and ZnttbPc in 5% F98 nanocapsule) included one band-pass interference filter (center wavelength: 650 nm, bandwidth: 60 nm) for excitation, one dichroic mirror (edge wavelength: 700 nm) for beam-splitting, and two long-pass interference filters (blocking band: 715 nm) for emission. The optical filter sets (Semrock, Rochester, New York) for laser pulse measurement included one band-pass interference filter (center wavelength: 650 nm, bandwidth: 60 nm) for excitation and one 50% beam splitter; no emission filter was used. The objective lens was 4× magnification, and the FOV size was ~3.2 mm in diameter.

### Synthesis protocols

The synthesis protocols of ADP(OH)_2_-Bottom in 5% F98 nanocapsules and ZnttbPc in 5% F98 nanocapsules were similar to the protocol of ADP(CA)_2_ nanocapsules adopted in our previous work^25,26^. Basically, 0.4 mg ADP(OH)_2_-Bottom dye or 1.2 mg ZnttbPc dye added with 4.8 mg TBAI were dissolved in 6 mL chloroform. 0.75 g pluoronic F98 was dissolved in 15 mL distilled water to get 5% F98 solution. The dye solution was then added dropwise to the 5% F98 solution with 600 rpm stirring. The mixture was under sonication (power: 40 Watts) for 4 mins to form nanocapsules. The mixture was stirred with 475 rpm overnight in a chemical hood until the chloroform evaporated totally. The solution was then filtered (membrane cut-off pore size: 450 μm) to obtain the purified sample.

### Sample configuration protocol of silicone phantoms

The protocol of making a silicone phantom was similar to that adopted in our previous work^24,25^. The silicone kit was purchased from Factor II Inc. (VST-50: VerSilTal Silicone Elastomer). The kit includes two major components: silicone elastomer and catalyst. Basically, 4 mg titanium dioxide (TiO_2_) was fully dissolved in 6 mL silicone catalyst by stirring for 20 mins and then mixed with 60 mL silicone elastomer. TiO_2_ functions as light scatters in the silicone phantom; the estimated absorption coefficient μ_a_ = 0.03, and the reduced scattering coefficient μ_s’_ = 3.5 cm^−1^ ^24,25,46^. The mixture was poured into a plastic container, and the silicone tube was inserted through the container. The amount of the poured mixture could be adjusted so that the silicone tube was imbedded at an appropriate depth. Then the sample was placed in a vacuum hood to remove the bubbles in the mixture. The silicone was solidified at room temperature for 12 hours. After that, the plastic container was peeled off, and the silicone phantom was ready to use.

## Supplementary information

Supplementary Information

## Data Availability

All data generated or analyzed during this study are included in this published article and its Supplementary Information.
