# Supplementary material for: An ICCD camera-based time-domain ultrasound-switchable fluorescence imaging system
Source: Sci Rep. 2019 Jul 22;9:10552. doi: 10.1038/s41598-019-47156-x (PMC6646316; doi:10.1038/s41598-019-47156-x)
Supplement: Supplementary file 1 — Supplementary Information [file 41598_2019_47156_MOESM1_ESM.pdf]

Supplementary Information

An ICCD camera-based time-domain ultrasound-switchable fluorescence imaging system

Shuai Yu, Tingfeng Yao, and Baohong Yuan*

Ultrasound and Optical Imaging Laboratory, Department of Bioengineering, The University of Texas at Arlington, Arlington, TX 76019, USA

Joint Biomedical Engineering Program, The University of Texas at Arlington and The University of Texas Southwestern Medical Center at Dallas, TX 75390, USA

**Corresponding author: Baohong Yuan, Mailing address: 500 UTA Blvd, Department of Bioengineering, Arlington, TX 76010, USA,*
Tel: +1-817-272-2917; FAX: +1-817-272-2251, E-mail: baohong@uta.edu

ADP(OH)$_2$-Bottom and ZnttbPc dye. The full name of ADP(OH)$_2$-Bottom is BF2-chelated [5-(4-hydroxyphenyl)-3-phenyl-1H-pyrrol-2-yl]-[5-(4-hydroxyphenyl)-3-phenylpyrrol-2-ylidene] amine[1]. Its absorption peak = 699 nm. The full name of ZnttbPc is zinc 2,9,16,23-tetra-tert-butyl-29H,31H-phthalocyanine, which is commercially available. Its absorption peak = 677 nm. Fig. S1 (a) and (b) show their chemical structures.

Fluorescence intensity and lifetime measurement of USF contrast agents as a function of temperature in the previous system. Similar to fluorescence intensity and lifetime measurement in the ICCD camera imaging system, we can also measure the I_{on}/I_{off} and τ_{on}/τ_{off} of the USF contrast agents in a PMT-based fluorescence lifetime measurement system. The measurement system was described in our previous work.[2] Briefly, a sub-nanosecond pulsed and nitrogen-pumped dye laser (peak wavelength: 655 nm) was used as the excitation source. An optical alignment system was adopted to collimate the fluorescence light and one band-pass interference filter (center wavelength: 711 nm, bandwidth: 25 nm; Semrock, Rochester, New York) was used to block the excitation light. A photomultiplier tube (PMT) was adopted as the detector and a multichannel oscilloscope was used for data acquisition. 3 mL agent solutions were prepared in a quartz cuvette submerged in a water bath via a small transparent glass container. The water temperature was controlled via a temperature controller, a heater, and a temperature sensor. Two agents were tested: 1) ADP(OH)$_2$-Bottom in 5% F98 nanocapsules and

2) ZnttbPc in 5% F98 nanocapsules. Fig. S2 (a) shows the normalized fluorescence intensity and lifetime change of $ADP(OH)_2$-Bottom in 5% F98 nanocapsules over a temperature rise. Likewise, Fig. S2 (b) shows the same information for ZnttbPc in 5% F98 nanocapsules. The result is similar to that from the ICCD camera imaging system measurements. $ADP(OH)_2$-Bottom in 5% F98 nanocapsule has a I_{on}/I_{off} ratio (I_{on}/I_{off} = ~ 24 folds), and its fluorescence lifetime slightly changes as a function of temperature (τ_{on}/τ_{off} = ~ 1.07, τ_{on} = 1.48 ns); its T_{th} = ~ 31°C. ZnttbPc in 5% F98 nanocapsule has both significant intensity and lifetime changes (I_{on}/I_{off} = ~ 4200 folds, τ_{on}/τ_{off} = ~ 11.23 folds, τ_{on} = 2.97 ns); its T_{th} = ~ 28°C.

Different lifetime of fluorescence and laser pulse on ICCD images. In this work it shows that the laser pulse can be much shorter (usually its lifetime < 500 ps) than some fluorescence pulses that have long fluorescence lifetimes (> 1.0 ns). In this section we demonstrated that this ICCD camera imaging system was capable of measuring different lifetimes on the same FOV. Fig. S3 (a) left shows the experiment sample setup. A silicone phantom (μ_s' = 3.5 cm^{-1}, μ_a = 0.03 cm^{-1}, total thickness Z = 3 mm) imbedded with a silicone tube (*I.D.* = 760 μm, Z1 = Z2 = 1.5 mm) was placed on a plastic Petri dish, and they were placed on the microscope stage for imaging (with a 4× objective lens). When the laser beam was focused on the bottom surface of the phantom, near the tube, the plastic Petri dish gave laser reflection and generated autofluorescence on the pathway. CCD images captured the corresponding signal spot (the round red spot in Fig. S3 (a) left). This signal should have a short lifetime. Meanwhile, the silicone tube was filled with ZnttbPc in 5% F98 nanocapsule. The Petri dish was filled with hot water at 40 °C so that the phantom was heated and the contrast agent inside the tube was switched on and presented a long lifetime. CCD images also captured the scattered fluorescence signal from the tube (the green line in Fig. S3 (a) left). Fig. S3 (a) right shows one real example image from the experiment. Fig. S3 (b) shows a series of CCD images captured at different gate delay t; t was counted from the start time of the gate, and the gate width was fixed at t_w = 300 ps as previously (seen in Fig. 1 (c)). Fig. S3 (b) clearly shows that the round beam spot appeared first but disappeared fast and that the fluorescence in the tube appeared later but disappeared much more slowly because of its long lifetime. Fig. S3 (c) shows the different lifetimes measured when different ROIs were selected. ROI 1 was selected on the round beam spot, so it showed a relatively short lifetime of laser leakage plus plastic autofluorescence. Its decay time (down to 37%, without deconvolution from impulse response function (IRF), same below) was ~ 0.49 ns. On the other hand, ROI 2 was

selected on the tube so it showed a long lifetime of fluorescence signal. Its decay time was ~ 3.87 ns.

USF signal lifetime measurement. In this study it shows that it is feasible to select a proper time-gated window of signal acquisition to improve SNR in USF imaging. This is based on the assumption that the USF signal has a longer lifetime than that of the background. Although it was shown that both $ADP(OH)_2$-Bottom nanocapsule and ZnttbPc nanocapsule demonstrated a long lifetime when the samples were tested in the cuvette in a high temperature environment (i.e., $T > T_{th}$), a question remained whether the USF signal itself had a long lifetime. In the previous section, we assumed that the USF signal came from the contrast agents that were thermally "switched-on" due to a temperature rise by FU heating. In this experiment, we measured the fluorescence lifetime of a USF signal, in order to demonstrate whether the "ultrasonically switched-on" fluorescence had a lifetime similar to that of the "thermally switched-on" fluorescence. Fig. S4 (a) shows the sample configuration and the experimental setup. In this experiment, we tested the USF signal from $ADP(OH)_2$-Bottom in 5% F98 nanocapsule. The silicone tube was filled with $ADP(OH)_2$-Bottom in 5% F98 nanocapsule, and the cuvette was filled with ZnttbPc in 5% nanocapsule. Thus, the background had a strong-intensity but short-lifetime fluorescence. The FU transducer was focused on the silicone tube for acquiring the USF signal. We adopted a long exposure time (3 s) to get the USF signal. The experimental setup was basically the same as that in Fig. 6 (a), Case 2, except that the FU transducer did not scan across the tube but acquired the USF signals from the tube at different time-gated windows.

This experiment followed three steps. In step 1, the ICCD camera measured the IRF of the system (when the cuvette and tube were first filled with water). Fig. S4 (b) presents the measurement principle, which is the same as that in Fig. 1 (c). Here, the gate width t_w = 400 ps, the delay time interval Δt = 200 ps, and the measured gate time range t = 0 – 10 ns. In Fig. S4 (c), the solid blue line represents the normalized IRF of the system (i.e., the measured excitation laser pulse). In step 2, the system measured the strong-intensity background fluorescence (BF) pulse from the cuvette filled with ZnttbPc in 5% nanocapsule, which is the solid red line (normalized) in Fig. S4 (c). Its calculated lifetime is 0.26 ns (function y1, dash red line in Fig. S4 (c)). Step 3 repeated the measurement in step 2, with the difference that a FU signal (Vpp = 130 mV, FU exposure time = 400 ms) came in. The FU signal was synchronized with each camera frame

(camera exposure time = 3 s) that captured the fluorescence pulse within the designed time-gated window as in step 2. Thus, in step 3, the measured fluorescence pulse represents the BF pulse (from the cuvette) plus the USF pulse (from the tube). Then, this measured pulse in step 3 was subtracted from the BF pulse in step 2; the remainder was only the USF pulse from the tube. In Fig. S4 (c), the solid green line represents the normalized USF pulse after subtraction. Its calculated lifetime is 1.10 ns (function y2, dash green line in Fig. S4 (c)). The result shows that the USF signal has a relatively long lifetime compared with that of the background (τ = 1.10 ns > 0.26 ns). It also shows the USF signal lifetime of ADP$(OH)_2$-Bottom nanocapsule is similar to the lifetime of the nanocapsule (τ = 1.02 ns as seen in Fig. 1 (f)) in a high-temperature (i.e., T = 40 °C > T_{th} = 31 °C) water-bath environment.

References

1 Bandi, V. *et al.* Excitation-Wavelength-Dependent, Ultrafast Photoinduced Electron Transfer in Bisferrocene/BF2-Chelated-Azadipyrromethene/Fullerene Tetrads. *Chemistry–A European Journal* **19**, 7221-7230 (2013).

2 Cheng, B. *et al.* Development of ultrasound-switchable fluorescence imaging contrast agents based on thermosensitive polymers and nanoparticles. *IEEE Journal of Selected Topics in Quantum Electronics* **20**, 67-80 (2014).

(a)

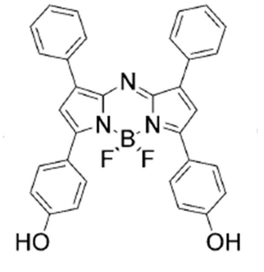

ADP(OH)$_2$_Bottom

Absorption peak = 699 nm

(b)

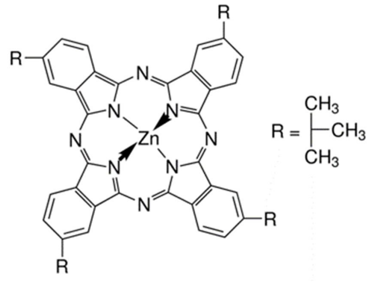

ZnttbPc

Absorption peak = 677 nm

Figure S1. (a) Chemical structure of ADP(OH)$_2$-Bottom. (b) Chemical structure of ZnttbPc.

(a) (b)

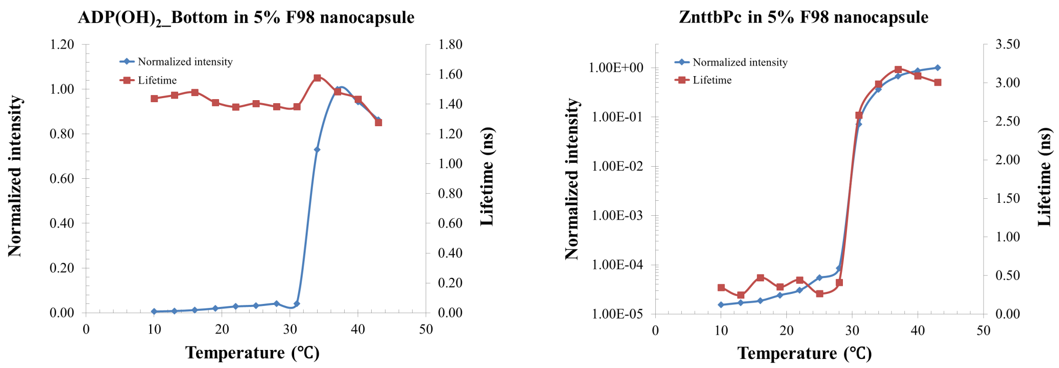

Figure S2. (a) Normalized fluorescence intensity and lifetime change of $ADP(OH)_2$-Bottom in 5% F98 nanocapsules over a temperature rise. (b) Normalized fluorescence intensity and lifetime change of ZnttbPc in 5% F98 nanocapsules over a temperature rise.

(a)

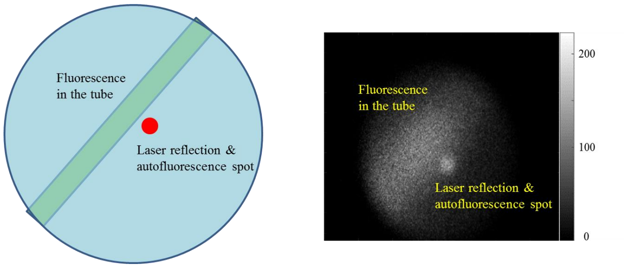

(b)

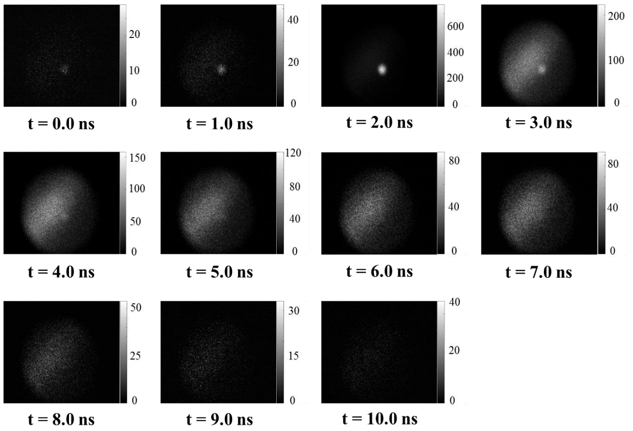

(c)

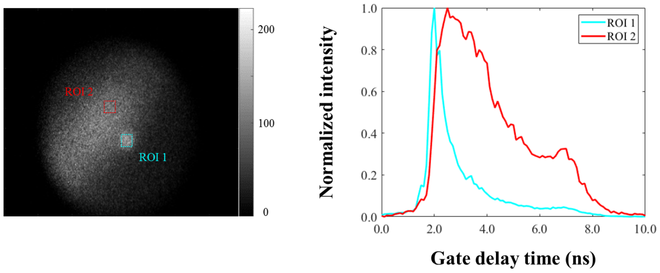

Figure S3. (a) Experiment sample set-up (left plot) and one real example image from the experiment (right plot). (b) A series of CCD images captured at different gate delay t. (c) Different measured lifetimes (seen in the right plot) when different region of interest (ROI) was selected: ROI 1 was selected on the round beam spot and ROI 2 was selected on the tube (seen in the left plot).

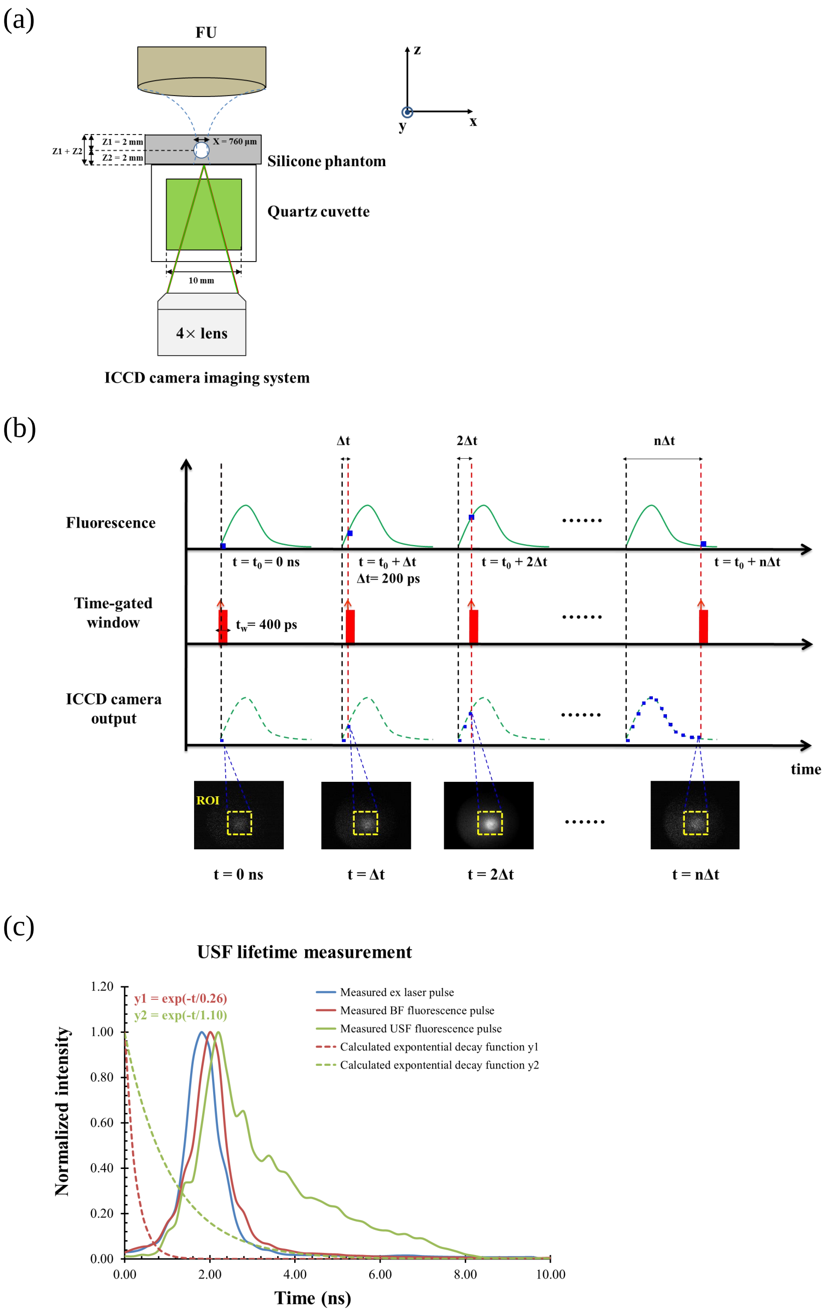

Figure S4. (a) Sample configuration and experiment set-up. (b) Principle of measuring the fast fluorescence decay signal after excited by a short laser pulse via the gated ICCD camera. Note t_w = 400 ps and Δt = 200 ps here. (c) The measured lifetimes of the background fluorescence pulse, as well as the USF pulse which came from $ADP(OH)_2$-Bottom in 5% pluoronic F98 nanocapsule in the tube.
